# Autoantibodies against BAFF, APRIL or IL21 - an alternative pathogenesis for antibody-deficiencies?

**DOI:** 10.1186/s12865-017-0217-9

**Published:** 2017-06-26

**Authors:** Marian-Christopher Pott, Natalie Frede, Jennifer Wanders, Lennart Hammarström, Erik-Oliver Glocker, Cristina Glocker, Fariba Tahami, Bodo Grimbacher

**Affiliations:** 1 Centre for Chronic Immunodeficiency, Medical Centre University Hospital, Medical Faculty of Freiburg, Freiburg, Germany; 2 Institute of Immunity and Transplantation, Royal Free Hospital, University College London, London, UK; 3 Department of Immunology, Karolinska Institutet, Stockholm, Sweden; 4 Institute of Medical Microbiology and Hygiene, University Medical Center Freiburg, Freiburg, Germany; 5 Institute of Laboratory Medicine, Brandenburg Hospital, Brandenburg Medical School, Brandenburg, Germany

**Keywords:** Autoimmunity, Autoantibodies, Cytokines, Primary antibody deficiency, Common variable immunodeficiency, Selective IgA deficiency, BAFF, APRIL, IL-21

## Abstract

**Background:**

The ability of anti-cytokine antibodies to play a disease-causing role in the pathogenesis of immunodeficiencies is widely accepted. The aim of this study was to investigate whether autoantibodies against BAFF (important B cell survival signal), APRIL (important plasma cell survival signal), or Interleukin-21 (important cytokine for immunoglobulin class switch) present an alternative mechanism for the development of the following primary antibody deficiencies (PADs): common variable immune deficiency (CVID) or selective IgA deficiency (sIgAD).

**Results:**

Two hundred thirty-two sera from patients with PADs were screened for autoantibodies against cytokines by ELISA. Statistical data analysis yielded a significant difference (*p* < 0.01) between the healthy donor sera and both PAD cohorts. The analysis was deepened by subdividing the patient collective into groups with distinct B cell phenotypes but no significant differences were found. For selected sera with notable high ELISA-read outs functional analysis ensued. Anti-BAFF and anti-APRIL antibodies were further examined by a B cell survival assay, whilst the functional relevance of putative anti-IL-21 autoantibodies was investigated by means of a STAT3 phosphorylation assay. However, the results of these experiments revealed no discernible functional effect.

**Conclusion:**

Whilst statistical analysis of ELISA results showed significant differences between patients and healthy controls, in our set of patients functional tests yielded no evidence for an involvement of autoantibodies against BAFF, APRIL, or IL-21 in the pathogenesis of CVID or sIgAD.

## Background

In recent years the role of autoantibodies against cytokines in disease pathogenesis has received an increased amount of attention. The existence of cytokine autoantibodies has been known for more than 30 years, but their significance in disease pathogenesis remains uncertain for many patients. In the context of viral infection [1], malignancy, extensive trauma, and therapy with recombinant proteins [2, 3], autoantibodies are mostly regarded as an epiphenomenon rather than disease-causing. Yet lately several studies were able to link autoantibodies against cytokines directly to disease pathogenesis: Kitamura et al. identified antibodies against GM-CSF in pulmonary alveolar proteinosis (PAP), which, by impairing alveolar macrophages, affected the immune-response [4]. Similarly, anti-erythropoetin antibodies have been found in patients with pure red-cell aplasia (PRCA) [5, 6] and many more have been described in literature [7–9]. Furthermore, anti-IL6 antibodies were discovered in a patient with recurrent staphylococcal cellulitis [10] and Kampmann et al. was able to show, that antiIFN-γ antibodies in patients resulted in a predisposition to mycobacterial diseases [11]. In view of these findings, autoantibodies against cytokines have sparked a special interest in the field of immunodeficiencies [7, 12].

Within the group of primary immunodeficiencies (PID), primary antibody deficiencies (PAD) constitute the lion’s share with approximately 50–70% [13]. While most PIDs are caused by single gene mutations, the underlying pathogenesis of PAD still remains poorly understood [14]. For common variable immunodeficiency (CVID), the most prevalent symptomatic PAD, several mutations have been described [15–19], although they only account for approximately 25% of CVID [17, 20]. In other PADs such as selective IgA Deficiency (sIgAD) no causal mutations have been found to this day [21]. However, in sIgAD several linkage studies were able to demonstrate an association to HLA-DQ/DR loci [22] as well as IFIH1, a locus associated with type 1 diabetes and systemic lupus erythematodes [23], strongly suggesting an autoimmune origin in CVID/sIgAD. Adding to this, autoimmune manifestations are frequent in PAD patients [24, 25].

Combining the growing relevance of autoantibodies against cytokines with the putative autoimmune etiology of primary antibody deficiencies, in this study we aimed to investigate whether autoantibodies directed against key-immunocytokines, were able to impair the maturation of an antibody response and ultimately culminating in a hypogammaglobulinemic phenotype.

The cytokines, which were investigated in this study, were chosen based on their role in the immune system. The presence of the antibody will prevent the cytokine to bind to its receptor and will thereby block further signaling.

BAFF and its homologous counterpart APRIL belong to the TNF-family of cytokines [26] and play a key role in the differentiation as well as the homoeostasis of the B cell- and plasma cell pool [27–29]. Both have been extensively studied in the context of CVID: BAFF receptor mutations have been described to cause CVID [15, 16] and BAFF and APRIL dysregulation have been reported in CVID patients [29, 30]. Considering that anti-cytokine autoantibodies have been reported to mimic the phenotype caused by a mutation of the respective cytokine, we picked BAFF and APRIL as appropriate candidate cytokines to investigate. IL-21 was chosen due to its significance in the regulation of B cell development as well as its role in the process of class-switching. Parrish-Novak et al. were able to show that IL-21 was key in activating the proliferation and differentiation of B cells into memory B cells and plasma cells [31, 32], whilst Okazi et al. reported that IL-21-deficient transgenic mice had markedly reduced serum levels of IgG1-3. Moreover, IL21-/IL4-deficient mice presented with a CVID phenotype [33–35], making it a particularly interesting cytokine.

Following up on the aforementioned studies, we screened for inhibitory autoantibodies against BAFF, APRIL and IL-21 in over 200 PAD patients. Overall, several significant differences were detected between PAD patients and healthy controls, but the absolute OD values of detected autoantibodies were low and, most significantly, sera positive for auto-antibodies failed to show neutralizing activity. Thus, no conclusive evidence for an involvement of autoantibodies against BAFF, APRIL or IL-21 in the pathogenesis of PAD could be established in our study.

## Results

### Screening for autoantibodies by ELISA

In order to search for autoantibodies, the ELISA-method was chosen, due to its high sensitivity as well as its capability to screen large numbers of samples in a time- and cost-efficient manner. Since all reported autoantibodies against cytokines have been of the IgG isotype [12, 36], we focused our search on this isotype only.

After having established a screening ELISA able to detect anti-cytokine IgG immunoglobulin, healthy donor sera and patient sera were analysed. Each sample was analysed three times. Samples were considered positive if the read-out was above the cut-off value, defined as the healthy donor mean + 2 standard deviation.

For the anti-BAFF ELISA 68 healthy donor sera and 174 PAD-patients were investigated. The PAD-patients consisted of 52 CVID-patients and 118 patients with sIgAD. After screening, 18 samples of the CVID group, and 52 of the sIgAD group produced a read-out higher than the cut-off.

For the APRIL-ELISA, a total of 41 healthy donor sera and 232 patient sera (113 CVID, 119 sIgAD) were screened. The results identified 27 CVID sera and 27 sIgAD sera with optical densities (OD) above the cut-off value. The anti-IL-21 ELISA included a total of 67 HD sera and 206 patient sera (83 CVID, and 118 sIgAD). Ten CVID patients and 52 sIgAD patients had values above the defined threshold.

**Fig. 1:**
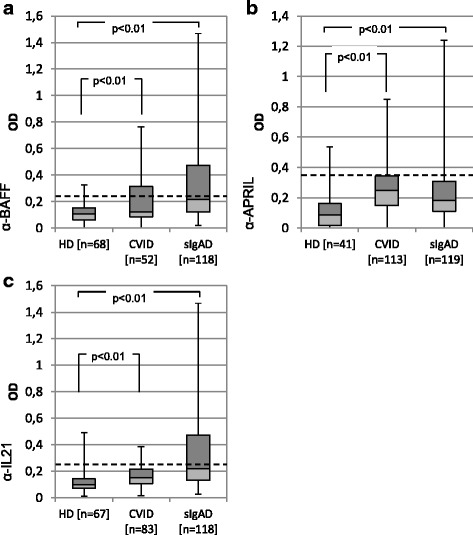
Results of Screening-ELISA. In this Box-and-Whiskers-Plot the bottom and top of the box represent the first and third quartiles of the dataset, the band inside the median, and the whiskers show the minimum and maximum values. In Panel (**a**), anti-BAFF ELISA read-outs show a significant difference between healthy donors and the CVID and sIgAD cohort. Similar results were obtained with anti-APRIL-ELISA (Panel **b**) and anti-IL-21 ELISA (Panel **c**). The dashed line marks a cut-off value for each ELISA, defined as the healthy donor mean + 2 standard deviation, above which samples were considered positive

The screening results can be viewed in Fig. 1. Data analysis with the t-test showed a statistically significant difference (*p* < 0.01) between the healthy donor groups and CVID- and sIgAD patients for all three cytokines.

### Subgroup analysis

**Fig. 2:**
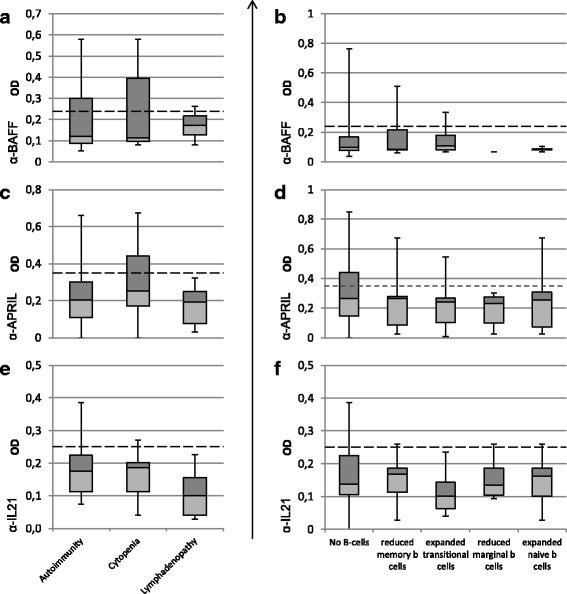
Subgroup analysis. Panel (**a**) shows the results of the BAFF-ELISA assorted by clinical characteristics. Comparing the distribution of these data subsets with the results of the healthy donor cohort was unable to show a significant difference. In Panel (**b**) the BAFF-ELISA results are sorted according to their B cell phenotype. In Panel (**c**) and (**d**) the results of the APRIL-ELISA are grouped by clinical manifestations and (**b**) cell phenotypes respectively. Panel (**e**) and (**f**) show the subgroups analysis for IL-21. The statistical analysis for none of the above yielded a significant result

Investigating whether the patient groups with high read-outs in the ELISA shared common characteristics, we sorted the patient pool depending on their cellular B cell phenotype and clinical manifestations, in particular autoimmune manifestations, cytopenia, and lymphadenopathy [26, 29, 37, 38]. The results can be viewed in Fig. 2 Panel a, c, and e. Following the EURO Classification Trial [39], B cells were subdivided into decreased numbers of total B cells (<1% CD19+), decreased switched memory B cells (<2% IgM- IgD- CD27+) and expanded transitional B cells (>9% CD38hi IgM hi CD27-). Further subgroups were defined as decreased marginal zone B cells (<2.5% IgM + IgD+ CD27+) and expanded naive B cells (>85% CD19+ IgM + IgD+ CD27-) (see Fig. 2 Panel b, d and f).

Considering that blocking the BAFF- and APRIL receptor BCMA led to a marked reduction of peripheral B cells [40] and that BAFF knock-out mice have markedly reduced mature B cells [41] as well as marginal zone B cells [37, 42], patients exhibiting these cellular characteristics were of particular interest. Furthermore, patients with expanded T1-transitional B cells were investigated, since a developmental block at the transitional B cell stage had been reported in BAFF receptor-mutated humans [15, 16] and BAFF knock-out mice [37, 43, 44]. However, the statistical analysis between these subgroups yielded no significant difference at either anti-cytokine autoantibody.

### Functional analysis

In order to address the ambiguity surrounding the functional significance of elevated autoantibody titres, we set up an array of functional tests to assess the effects that autoantibodies might have on the immune system. A specific assay was established for each cytokine. Based on the property of BAFF and APRIL to promote the survival of B cells [37, 40, 45–47] a survival assay was devised. To detect dead cells, FACS analysis with DAPI-staining was chosen. Pre-testing yielded an ideal incubation period of 9 days.

The samples selected for functional analysis had to comply with several criteria: Firstly, an ELISA read-out above the cut-off was required. Polyreactive sera, i.e. sera with high read-outs in every ELISA, were omitted. Secondly, the cellular phenotype should match with the involved cytokine. In case of BAFF, we would expect low B cell numbers, expanded transitional B cells, and low BAFF levels. Lastly, a known mutation in BAFF should be excluded.

**Fig. 3:**
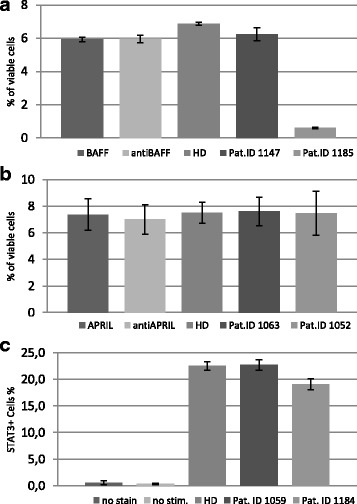
Functional Analysis. In Panel (**a**) and (**b**) the results of the B cell survival assay are displayed. In Panel (**a**) viable (**b**) cells (CD19+,DAPI-) are shown after 9 days-incubation with patient sera, which were selected for their high anti-BAFF-ELISA results. Pat.1185 had received Rituximab-treatment prior to serum withdrawal; hence the results were not conclusive. Panel (**b**) shows the amount of viable (**b**) cells after the addition of sera with putative anti-APRIL antibodies. The results show no significant difference. In Panel (**c**) the results of the STAT3 phosphorylation assay are shown. PBMCs were incubated with IL-21 as well as selected healthy donor and patient sera. pSTAT3 was measured by FACS analysis. No marked disparity was observed between patients and healthy donors

After the incubation period, viable cells were counted. Each functional experiment was repeated twice. The results can be seen in Fig. 3a and b. Since it was known that the selected patient sera had low BAFF-levels, we presumed that the cell culture with HD serum would contain the highest concentration of BAFF and therefore retain the largest amount of viable cells after 9 days. This prediction was confirmed by our experiment. If the patient with ID#1147 had anti-BAFF antibodies able to block BAFF signalling, we would expect a decreased amount of living cells. The results however, showed a similar cell count to the HD control, rendering functional antibodies in this sample unlikely. In the case of patient ID#1185, a marked reduction of living B cells was observed, making this result compatible with the presence of BAFF autoantibodies. However, after examining the patient’s medical history, an anti-B cell directed therapy with the monoclonal anti-CD20 antibody rituximab was reported. This detail sheds a different light on our results and makes the presence of anti-BAFF autoantibodies an unlikely reason for the observed B cell deficiency.

Contrary to the BAFF survival assay, APRIL levels in patients were unknown, hindering prediction on the outcome of the experiment. Although APRIL levels are reported to be high in patients with CVID [30], patients with normal or reduced APRIL levels are not uncommon [30].

High levels of APRIL promote B cell survival and in case of autoantibodies against APRIL a notable decrease in live cells would be expected. The experimental results (Fig. 3b) however, do not match our expectations and thus the existence of functional autoantibodies seems improbable.

To investigate the functionality of supposed anti-IL21 antibodies, a STAT3-phosphorylisation assay was used. To detect the phosphorylated STAT3 (pSTAT3) by FACS-analysis we followed the STAT3-phosphlow protocol (BD Pharmigen) according to the manufacturer’s instructions. When IL-21 binds to its receptor, a signaling cascade is started, which includes the phosphorylation of STAT3. Autoantibodies against IL-21 should therefore lead to a decrease in pSTAT3.

In Fig. 3c we show that the difference between the healthy donor pSTAT3 and the ones of our patients is only marginal, rendering a functional involvement of putative autoantibodies negligable.

## Discussion

Although the screening-ELISAs returned a considerable amount of positive output with a statistical analysis hinting a significant difference (*p* < 0.01), we were not able to demonstrate any functional effect of those autoantibodies, which seemed to be elevated just above the baseline without any functional impact. This hypothesis is supported by the observation that often a single sample had several anti-cytokine antibodies elevated (polyreactivity), which may be an intrinsic feature to patients with PADs.

Compared to commercially available ELISA kits, the sensitivity and specificity of our in-house established ELISA tests was unknown. Even though positive and negative controls were implemented, several altering factors have to be taken into account: The most crucial aspect were non-specific bindings of antibodies to a natural occurring antigen (e.g. plastic), potentially masking correct binding with the coating cytokine. Whilst our ELISA protocol accounted for some of these disturbances by blocking the wells with BSA and duplicating each step on a non-coated well plate, inadequacies were difficult to quantify. Furthermore, it has to be considered that some autoantibodies may have been bound to its respective cytokine, thus not detectable by ELISA.

## Conclusion

Addressing the initial hypothesis, our experimental data suggests that antibodies against the cytokines BAFF, ARIL, and IL-21 do not play a role in the pathogenesis of primary antibody deficiencies. Although our ELISA screening results pointed towards elevated anti-cytokine antibody titers, our functional analysis demonstrated no relevant involvement of these putative anti-cytokine antibodies in the etiopathophysiology of antibody deficiencies. Thus, the existence of autoantibodies cannot be revoked; their role in the formation of disease however, remains elusive. It is in particular the clinical heterogeneity of PADs such as CVID that should encourage scientists to consider alternative pathomechanisms, and an autoimmune origin of antibody deficiencies remains a promising area of research.

## Methods

### Patient cohorts and cell culture

The study followed the Declaration of Helsinki and all subjects provided informed consent for participation. Patient serum samples (*n* = 232) were collected from the Institute of Immunity and Transplantation at the Royal Free Hospital London and from the Karolinska University Hospital Stockholm. 115 patients had CVID, and 116 had sIgAD. Patients were diagnosed according to the criteria established by the European Society for Immunodeficiencies [48]. Upon receipt, samples were aliquoted and frozen until analysis. Informed consent was obtained from all patients and approval was granted by the local ethics committee.

### Enzyme-linked immunosorbent assay (ELISA)

Maxisorp 96-well ELISA plates (Maxisorp; Nunc) were coated with the recombinant cytokines by incubation overnight at 4 °C at their respective concentration. They were then washed (PBS-Tween 0.05%) and incubated for 1 h with the same buffer supplemented with 5% nonfat milk powder, in order to block free binding sites. After being washed twice, the patient sera were added and incubated 2 h at room temperature. Thereafter plates were thoroughly washed and Fc-specific HRP-conjugated IgG-fractions of polyclonal goat antiserum against human IgG (Peprotech) were added. After an incubation time of 1 h at room temperature, the plates were washed, substrate TMB was added and absorption was determined in an ELISA reader at 450 nm. For the positive control we used polyclonal antibodies against the cytokine (Peprotech), which were produced from sera of goats immunized with the specific recombinant human cytokine. Negative controls were carried out by adding the detection antibody to a non-incubated plate. To quantify unspecific bindings each serum sample was incubated concurrently in a coated as well as a non-coated well.

### STAT3 phosphorylation assay

Peripheral blood mononuclear cells (PBMCs) from healthy donors were isolated by using density gradient centrifugation and then washed in PBS (Sigma, US). 10^5^ PBMCs were re-suspended in RPMI 1640 (Sigma-Aldrich).

Prior to adding 50 ng/ml Il-21, patients’ sera were added to the cell culture at a 1:200 dilution and incubated for 30 min. After another 20 min the cells were fixed with 200 μl Cytofix Buffer (BD Bioscience). Once the cells were centrifuged, the supernatant was discarded and 500 μl Permbuffer III (BD Bioscience) added to permeabilize the cells. After thorough washing, the cells were stained with 5 μl of a FITC p-STAT3 antibody and incubated protected from light for 60 min. Lastly the cells were centrifuged, the supernatant discarded and the pellet resuspended in 500 μl Stain-Buffer (BD Pharmingen™) for FACS analysis.

### Survival assay

Using a 24-well plate (Thermo Fisher Scientific) 10^5^ PBMCs were cultivated in 1 ml cell medium, consisting of RPMI 1640 supplemented with 10% heat-inactivated FBS and Streptavidin. Cells were then stimulated with BAFF at a concentration of 0.2 μg/ml. Selected patient sera were then added at a dilution of 1:200.

An incubation-period of 9 days was found to provide the best ratio of dead/alive cells. Cells transferred into FACS-Tubes, washed using 3 ml of FACS-Buffer and then centrifuged for 8 min at 1200 rpm at 4 °C. After discarding the supernatant, the step was repeated. Binding buffer was added and the cells stained with 5 μl DAPI und 5 μl APC anti-CD19. After a 15 min incubation period away from light, the cells were analyzed by FACS (LSR II flow cytometer). The data was evaluated using FACSDiva Software (BD Biosciences).

### Statistical analysis

In line with literature, we assumed a normal distribution of immunoglobulins [49], allowing us to execute a parametric statistical analysis using a t-test. Each PAD-group was compared to the healthy donor data set. Two-tailed *p*-values < 0 · 01 were considered statistically significant.

## Abbreviations

APRIL: A proliferation inducing ligand
APS-I: Autoimmune polyendocrinopathy I
BAFF: B cell activating factor
CMC: Chronic mucocutaneous candidiasis
CVID: Common variable immunodeficiency
IFN-gamma: Interferon gamma
IL-21: Interleukin 21
sIgAD: selective IgA deficiency
